# You gotta walk the walk to talk the talk: protocol for a feasibility study of the Happy Older Latino Adults (HOLA) health promotion intervention for older HIV-positive Latino men

**DOI:** 10.1186/s40814-023-01262-w

**Published:** 2023-02-28

**Authors:** Daniel E. Jimenez, Elliott R. Weinstein, John Batsis

**Affiliations:** 1 Department of Psychiatry and Behavioral Sciences, University of Miami Miller School of Medicine, Miami, USA; 2 Department of Psychology, University of Miami, Miami, USA; 3 Division of Geriatric Medicine, Department of Medicine and Department of Nutrition, Gillings School of Global Public Health, University of North Carolina, Chapel Hill, USA

**Keywords:** Latinos, Older adults, Health promotion, HIV, Cardiometabolic risk

## Abstract

**Background:**

Older Latinos living with the human immunodeficiency virus (HIV) have been disproportionately affected by the epidemic and experience compounded health disparities that have deepened over time. These health disparities are largely related to lifestyle and are either preventable or amenable to early detection or intervention. Despite existing resources to deliver an intervention to reduce this compounded health disparity, there is little information on the effects of health promotion interventions on indices of cardiometabolic risk in midlife and older Latinos living with HIV. The Happy Older Latinos are Active (HOLA) intervention is an innovative health promotion program that is uniquely tailored to meet the diverse needs and circumstances of older Latinos with HIV. The goal of this manuscript is to describe the protocol of a feasibility study of the HOLA health promotion intervention for older HIV-positive Latino men.

**Methods/design:**

HOLA, which is informed by Behavioral Activation and Social Learning theory is a community health worker (CHW)-led, multicomponent, health promotion intervention consisting of: (1) a social and physical activation session; (2) a moderately intense group walk led by a CHW for 45 min, 3×/week for 16 weeks; (3) pleasant events (e.g., going to brunch with friends) scheduling. Eighteen community dwelling Latinos living with HIV aged 50+ will be recruited for this feasibility study adapting the HOLA intervention. Participants will be assessed at three time points (baseline, post-intervention, and 3 months post-intervention) on measures of cardiometabolic risk factors (waist circumference, dyslipidemia, hypertension, and glucose), psychosocial functioning, and health-related quality of life.

**Conclusions:**

If HOLA can be delivered successfully by CHWs, then the scalability, accessibility, and potential for dissemination is increased. Additionally, this study will inform feasibility and identify modifications needed in the design of a larger hypothesis testing study.

**Trial registration:**

Clinicaltrials.gov Identifier: NCT 03839212. Date of Registration: 8 February, 2019.

## Background

Due to the changing landscape of the human immunodeficiency virus (HIV) epidemic, older adults are living with HIV at rates higher than ever before. In 2018, the prevalence of diagnosed HIV infection in the USA was 374.6 per 100,000 population with an increased number of estimated cases affecting older Americans over the age of 50 [1]. Even though HIV is no longer a death sentence due to the development of effective antiretroviral therapy (ART) and other HIV focused therapeutics, significant disparities in treatment outcomes persist among certain demographics. In general, HIV continues to not only affect racial/ethnic minorities at higher rates, but also gay, bisexual, and men who have sex with men (MSM) compared to their cis-gender heterosexual white counterparts [2]. Therefore, research must look to the intersections of age, race, ethnicity, and sexual identity in developing interventions that may best support those most at risk for poor HIV-related treatment outcomes.

Significantly more people living with HIV (PLWH) are living into older adulthood demonstrating a growing need for HIV-focused research and treatment examined through an aging lens. According to 2018 CDC data, more than half of PLWH in the USA are over the age of 50 years [1] with predictions that by the start of 2021 almost 70% of PLWH in the USA will be at least 50 years old [3]. Older adults are often diagnosed with HIV much later (~ 4.5 years after infection) indicating more advanced disease progression associated with the epidemic among this age group [1]. Additionally, for PLWH in the USA aged 55 and above, while 90% knew their HIV status, only 64% were virally suppressed and barely more than half (57%) were actively engaged in care, suggesting that the UNAIDS “90-90-90” goals toward ending the HIV epidemic by 2050 [4] are currently out of reach among older adults living with HIV (OALWH) [1].

Latinos have been disproportionately affected by HIV since the origins of the epidemic, and that increased vulnerability has deepened over time. Despite representing less than 20% of the entire U.S. population, Latinos make up 27% of new HIV diagnoses and account for over 20% of the national prevalence [5]. More specifically, Latino sexual minority men (LSMM) are responsible for almost 25% of new HIV infections among all gay, bisexual, and MSM identified individuals [2] despite Latino men making up less than 9% of the entire U.S. population [6]. These disparities persist beyond just HIV incidence with Latino identified PLWH facing additional challenges with their HIV treatment care due to suboptimal rates of viral suppression [7, 8] and ART adherence [9] compared to their non-Latino SMM peers.

Unsurprisingly, these racial/ethnic disparities become significantly more observable among older adults. Older Latinos in the USA are slightly more than 5 times more likely to acquire HIV [10] and are more likely to have an AIDS diagnosis, detectable viral load, and poorer treatment adherence compared to their age-matched white non-Latino counterparts [11, 12]. Additionally, older Latinos seem to exhibit mild to moderate cognitive impairment in learning, memory, and processing speed compared to their non-Latino white peers [12, 13]. It is likely that these intersecting social determinants (i.e., age, race/ethnicity, sexual identity) will continue as the HIV population ages and that many of these poor health outcomes will be felt most intensely by older Latinos.

Older Latinos living with HIV experience various physical health disparities due to their intersecting minority status that must be considered. In general, OALWH face increased rates of age-related comorbidities due to a phenomenon called accelerated aging [14, 15]. Data from a large observational cohort study of OALWH reported that over 77% of participants reported suffering from two or more health comorbidities in addition to HIV [16] with the average number of physical comorbidities per participants landing at three [17]. In particular, older HIV-positive Latino individuals are disproportionately affected with cardiometabolic diseases including metabolic syndrome (MetS), a precursor to diabetes, as well as cardiometabolic risk factors such as obesity and hypertension compared to their non-Latino older white peers [18–20]. Moreover, older Latinos living with HIV are more likely to be sedentary and not as actively engaged in pursuing changes in their physical activity compared to their non-Latino white counterparts [21]. Despite evidence of the “Hispanic Paradox^1^” [22], this lack of physical activity in combination with possible issues connected to ART medication and Hepatitis-C co-infection make older Latino adults living with HIV more likely to suffer from other complicating physical conditions like nonalcoholic fatty liver disease [23, 24] and cardiovascular issues compared to their non-Latino white counterparts.

Older Latinos living with HIV face elevated rates of mental health concerns in addition to these physical comorbidities. More generally, OALWH have documented rates of major depressive episodes anywhere between 18 and 52% [17, 25, 26], significantly higher than the 1–8% documented rates of depression among older adults in the general population [27]. Rates of social isolation in the general aging public (60 years or older) are estimated to be anywhere between 33% and 50% [28] with evidence suggesting that OALWH face social isolation more often than their age matched peers in the general population [29, 30]. Similarly, there is strong evidence to suggest that loneliness exponentially increases with age and one could predict OALWH bear even a greater amount of loneliness due to reduced social networks and ostracism [31, 32]. Additionally, loneliness and social isolation have been correlated with levels of morbidity and mortality comparable to more established biopsychosocial risk factors like obesity, sedentary behavior, smoking, and hypertension [28, 33, 34].

For OALWH, one of the biggest contributing factors to loneliness and social isolation is that of co-occurring stigma. While exact estimates of HIV stigma and age-related stigma in the USA are hard to calculate, OALWH must often navigate the dueling stigmas of HIV stigma, ageism, and stigma resulting from other possible marginalized identities [35–37]. It is possible that rates of stigma and loneliness seem to skyrocket in OALWH due to the increased likelihood that OALWH live alone and have limited and often inconsistent social networks [25, 37]. OALWH can face ostracism from the larger LGBTQ+ community and stigma due to the intersection of their age and HIV status compared to their non-infected peers which may in turn contribute to the increased levels of depression among this already vulnerable population [38]. Additionally, since familism and social cohesion are strong hallmarks of Latino culture [39, 40], Latino OALWH may experience the harmful effects of co-morbid stigma and social isolation more intensely than their non-Latino HIV-positive peers due to societal expectation that they be more connected to family as they age; however, more research must be conducted to determine the veracity of such a hypothesis.

As outlined above, compounded health disparities place older Latinos living with HIV at particularly high risk for diminished quality of life due to physical and mental health morbidity. These data underscore the public health importance of increased efforts to address the multiplicative and unequal burden of HIV, MetS, and diabetes shouldered by older Latinos. Therefore, based on this gap in the literature, there is a compelling need to develop and disseminate interventions that promote healthy living, combat social isolation, and improve HIV-related health outcomes among older Latinos living with HIV. The goal of this manuscript is to describe the protocol of a feasibility study of the Happy Older Latinos are Active (HOLA) health promotion intervention for older HIV-positive Latino men. Since this is a feasibility study, researchers will be focused on evaluating the feasibility of recruitment, retention, assessment procedures, and acceptability of an innovative application of an already established health promotion intervention, HOLA, to a new population, older Latino men living with HIV [41]. Also, in accordance with recommendations from biostatistical workgroups funded by NIH [42], this study will not powered to test a hypothesis. Rather, this study will serve as an initial step in establishing feasibility and acceptability of an approach that is intended to ultimately be used in a larger scale study. The specific aims of this study will be to:

1. Evaluate the feasibility of recruitment, assessment procedures, retention, acceptability, and implementation of HOLA in a sample of midlife and older Latinos with HIV.
2. Identify modifications needed in the design of a larger, confirmatory randomized controlled trial.
3. Explore changes in cardiometabolic risk factors (waist circumference, dyslipidemia, hypertension, and glucose), psychosocial functioning (depression and anxiety severity, social support), and health-related quality of life in a sample of midlife and older Latinos with HIV enrolled in the HOLA health promotion intervention.

## Methods

All study methods, protocols, and participant incentive structures were approved by the university’s Internal Review Board (IRB ID: 20181032). In addition, a SPIRIT checklist has been completed to serve as a brief, structured summary of the trial. See Table 1.

**Table 1:** SPIRIT Checklist

Data category	Information
Primary registry and trial identifying number	Clinicaltrials.gov Identifier: NCT 03839212
Date of registration in primary registry	8 February, 2019
Secondary identifying numbers	U54MD002266
Source(s) of monetary or material support	National Institute on Minority Health and Health Disparities (NIMHD)
Primary sponsor	University of Miami
Contact for public queries	Daniel Jimenez, Ph.D. (dej18@miami.edu)
Contact for scientific queries	Daniel Jimenez, Ph.D. (dej18@miami.edu)
Public title	The Happy Older Latinos Are Active (HOLA) Health Promotion Study in HIV-Infected Latino Men (HOLAHIV)
Scientific title	Preventing Cardiometabolic Disease in HIV-Infected Latino Men Through a Culturally Tailored Health Promotion Intervention
Countries of recruitment	USA
Health condition(s) or problem(s) studied	Human immunodeficiency virus (HIV), cardiometabolic risk
Intervention(s)	Happy Older Latinos are Active (HOLA): A 16-week multi-component, health promotion intervention
Key inclusion and exclusion criteria	Ages eligible for study: ≥ 50 years Sexes eligible for study: men Accepts healthy volunteers: no Inclusion criteria: older Latino (≥ 50 years); HIV infected but are virologically suppressed have documented risk of cardiometabolic disease. Exclusion criteria: diagnosis of diabetes, any neurodegenerative disorder, or dementia, or significant cognitive impairment; contraindications to physical activity; terminal physical illness; acute or severe medical illness that precludes safe participation.
Study type	Interventional Interventional study model: single group assignment Number of arms: 1 Masking: none (open label) Allocation: N/A
Target sample size	18
Recruitment status	Not recruiting
Recruitment Rate	6 participants per month
Primary outcome(s)	Number of eligible participants refusing to participate: 20% or less of eligible participants refusing to participate Retention rate: 85% or more of participants completing the post-intervention assessment Acceptability of intervention: 80% or more of sessions attended by participants
Key secondary outcomes	Change in cardiometabolic risk Change in psychosocial functioning Change in health-related quality of life
Statistical methods	Descriptive statistics with 95% confidence intervals; For continuous variables that are normally distributed: means and standard deviations. For continuous variables that are skewed: median and range; Categorical variables: summarized using counts and percentages.

### Participants

This feasibility study will enroll 18 Latino older (aged 50+) men living with HIV who will then be assigned to three intervention groups composed of 6 participants each. Although small, this sample size is consistent with similar feasibility studies focused on physical activity among older adults [43, 44] and will be large enough to establish feasibility of the intervention in a population of older Latino men living with HIV. Additionally, informed consent will be obtained from each participant prior to enrollment and all enrollees will be provided with an initial verbal summary of the study. Payments will be graduated so participants received $15 on the first visit, $25 on the second visit, and $35 on the third visit (total of honoraria = $75). Financial incentives have the potential to serve as undue inducements by diminishing peoples’ sensitivity to research risks or unjust inducements by preferentially increasing enrollment among underserved individuals. However, results from two low-risk randomized clinical trials indicate that there is no evidence from studies of participation in hypothetical or real randomized clinical trials that incentivizing enrollment is undue or unjust, suggesting that studies that offer participation incentives are not unethical [45]. For a detailed description of the inclusion/exclusion criteria, see Table 2.

**Table 2:** Inclusion/exclusion criteria

Inclusion criteria	Exclusion criteria
Latino (self-identified)	Have a diabetes diagnosis
Age 50+	Have a diagnosis of any neurodegenerative disorder or dementia (Parkinson’s disease, Alzheimer’s, vascular, frontotemporal dementia, etc.) or significant cognitive impairment as indicated by a Mini-Mental Status Exam score < 24
Male	Have contraindications to physical activity outlined in the American College of Sports Medicine standards
HIV infected but are virologically suppressed (viral load < 200 copies/mL)	Are unable to complete 10-m walk test
Volunteer informed consent	Currently residing in a nursing or group home
Expect to stay in Miami for the next 6 months	Have a terminal physical illness expected to result in the death within 1 year
Have documented risk of cardiometabolic disease.	Have an acute or severe medical illness that precludes them from safely participating in a health promotion intervention (e.g., progressive, degenerative neurologic disease, such as Parkinson’s Disease, multiple sclerosis, ALS; severe arthritis or orthopedic condition that would prevent participation in a physical activity program; lung disease requiring either oral or injected steroids, or the use of supplemental oxygen; New York Heart Association Class III or IV congestive heart failure, clinically significant aortic stenosis, history of cardiac arrest, use of a cardiac defibrillator, or uncontrolled angina; renal disease requiring the use of dialysis; cancer being actively treated with radiation or chemotherapy; myocardial infarction, CABG, or valve replacement within the past 6 months; serious conduction disorder, such as 3rd degree heart block; uncontrolled arrhythmia; pulmonary embolism or deep venous thrombosis within past 6 months; uncontrolled diabetes with recent weight loss, diabetic coma or frequent insulin reactions; stroke, hip fracture, hip or knee replacement, or spinal surgery in the past 6 months; receiving physical therapy for gait, balance, or other lower extremity training; severe, uncontrolled hypertension-systolic blood pressure > 200 mmHg and/or diastolic blood pressure > 110 mmHg)
	Are currently taking antidepressant medications in doses indicated for weight reduction.

### Recruitment

Participants will be recruited through two consent-to-contact databases with over 1200 participants each—one of people with HIV (recruited from the university-affiliated adult HIV clinic) and another focused on a community needs assessment (composed of HIV-negative and HIV-positive community dwelling adults). These databases include contact information, demographic information and data associated with HIV-related risk factors such as homelessness and psychological distress. Only participants who had indicated that they were HIV+ will be recruited from these databases. More information on how these consent to contact databases work can be found here [46].

### Conceptual framework

**Fig. 1:**
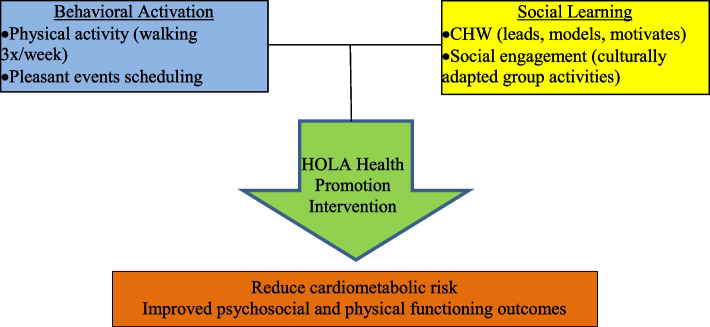
Conceptual framework

The conceptual model which serves as the foundation for the intervention (Fig. 1) was crafted to address comorbid depression and anxiety symptoms as well as both physical and psychosocial functioning of older Latinos. HOLA, is informed by Behavioral Activation (BA) [47] and Social Learning Theory (SLT) [48]. A major component of BA is scheduling activities into an individual’s day-to-day routine as a way of activating them out of a depressive episode. In HOLA, we incorporated components of BA in two ways. First, we encouraged participants to engage in a physical activity routine (i.e., an activity) and to schedule in pleasant events to their day-to-day (i.e., activity scheduling) to combat incident and recurrent episodes of depression and anxiety disorders as well as subdue symptom intensity [47]. As a complement, SLT’s tenets of reinforcement, observational learning, and enhanced self-efficacy are utilized to bolster participant engagement and success in the intervention [48]. The relationship between the participants and the community health worker (CHW) capitalizes on the personal relationship to motivate, model, and maintain health behavior change.

CHWs are an effective and culturally acceptable means of reaching the population with health information and motivating health behaviors [49, 50]. CHWs are lay community members who work almost exclusively in community settings and connect consumers to providers in order to promote health and prevent diseases among groups that have traditionally lacked access to adequate care [49]. CHWs are assumed to be effective because they possess an intimate understanding of community social networks and health needs; communicate in a similar language; and recognize and incorporate culture to promote health [49, 50]. The use of CHWs has emerged as a strategy to reduce or eliminate health disparities and is an important means of task shifting to enable efficient utilization of scarce mental health resources (see footnote below)^2^. Additionally, since engaging in health behavior change via physical activity is challenging, the HOLA intervention offers several opportunities for participants to be held accountable to their goals in the intervention. The CHW holds the individuals accountable, and individuals hold themselves accountable to the group, providing extra motivation to engage in the intervention. Accountability is an ideal way to help participants maintain their commitment, keep their energy and enthusiasm high and feel like they are not alone [51]. CHWs in this study will be trained in the HOLA intervention protocol and supervised by the senior author.

### Intervention

Happy Older Latino Adults (HOLA) is a multi-component health promotion intervention for midlife and older Latinos [42]. The first component consists of two manualized social and physical activation sessions. Prior to beginning the group walk phase, each participant will meet individually with a CHW for a 30-min physical and social activation session to (a) educate potential participants about the goals of the intervention; (b) provide information surrounding HIV/AIDS, cardiometabolic disorders such as diabetes and metabolic syndrome, how these physical conditions impact mental health, and ways they can improve their cardiometabolic health; (c) motivate participants to engage in physical activity; (d) increase participants’ social activities (e) identify potential obstacles that may interfere with meeting the demands of the intervention; and (f) brainstorm ways to overcome these obstacles. After week 8, participants will again meet one-on-one with the CHW for the second session so that they could discuss their own individual progress in relation to their physical and social activity goals.

The second component is centered around a group walk meant to facilitate both physical activity and social interaction between participants. This group walk will meet for 45 min, three times a week, for a total of 16 weeks. The group walk component was designed with interval training in mind and gradually increases in workload (defined by intensity, volume, and work/recovery cycle) over the course of the intervention. Each group walk will begin with 10 min of stretching and warm up, followed by 30 min of walking, and will end with 5 min of stretching/cool down. Each group walk will be led by the CHW and will be composed of six bilingual and monolingual Spanish-speaking participants.

The third component consists of scheduling pleasant events. During the cool down phase of each walking session, the CHW will ask each participant to identify a pleasant event that they intend to do with another person before the next meeting (e.g., going to brunch with friends). Individuals may choose to do this activity with another member of the group, with family, or with friends outside the group. Subsequent sessions will start with participants reporting on how effectively they implemented their pleasant event plan while the CHW and the group provide positive reinforcement and feedback. This component provides a means to generalize the intervention into the participants’ everyday lives and relationships. Participants will walk at a centrally located public park, which is owned and operated by the Miami-Dade County Parks and Recreation Department.

The fourth and final component of HOLA in the context of this feasibility study focuses on maintaining behavior change gleaned during intervention. Participants will engage in “booster” walking sessions, twice a month for 3 months post-intervention (starting the week after the 16-week program concluded) to capitalize on beneficial physical and mental health effects gained during the 16-week program. Encouraged by the prior literature, this maintenance phase was added to this feasibility study with the hopes of cultivating more sustained treatment effects over time [52]. A more in-depth overview of the HOLA intervention can be reviewed in the main protocol paper published by Jimenez and colleagues [41]. Adaptations made to the original HOLA intervention to make it specific to a sample of OALWH can be found in Table 3.

**Table 3:** Summary of adaptations made to original HOLA

Original HOLA	Adaptations made
Two social and physical activation sessions focused on education on depression and anxiety.	The focus of the social and physical activation sessions is to provide information surrounding HIV/AIDS, cardiometabolic disorders such as diabetes and metabolic syndrome, how these physical conditions impact mental health, and ways they can improve their cardiometabolic health.
Moderately intense group walk	No changes
Pleasant events scheduling	No changes
No maintenance phase	3-month maintenance phase

### Measures and analysis

This quasi-experimental feasibility study of an adapted health promotion intervention will examine feasibility of recruitment, assessment procedures, retention, acceptability, and implementation of HOLA in a sample of older Latinos living with HIV. In keeping with guidance from NIH funded biostatistical workgroups, this feasibility study was not designed to be powered to test a hypothesis [42]. Additionally, the proposed analysis of feasibility data parallels a similar structure employed in the first HOLA trial and comparable feasibility trials [41, 53]. For this study, successful recruitment will be defined as 100% of the targeted sample (*N* = 18) be enrolled and less than 20% of eligible subjects refusing to participate. Additionally, adequate retention will be characterized as 85% or more of enrolled participants completing all post-intervention assessments while acceptability will be defined as participants attending at least 80% of sessions. Finally, with goals of scaling up the intervention in the future, an established project evaluation questionnaire developed by investigators at the University of Miami will be used to pinpoint any modifications needed for the design of a larger, confirmatory randomized control trial. The questionnaire is made up of a series of closed ended yes/no questions, rating scales, and open-ended questions that allow for more qualitative data regarding participants’ opinions of the specific components of the overall intervention. Study measures will be administered at baseline, end of intervention, and 3 months post-intervention. Trained research assistants (RA) will administer all of the assessments.

### Feasibility outcomes

Study feasibility will be evaluated via participant recruitment, retention, and acceptability of the overall intervention. First, authors will measure study feasibility via recruitment and retention of eligible participants. For this study, successful recruitment will be defined as 100% of the targeted sample (*N* = 18) be enrolled and less than 20% of eligible subjects refusing to participate. Additionally, adequate retention will be characterized as 85% or more of enrolled participants completing all post-intervention assessments. Second, authors will assess participant acceptability of the intervention as another component of study feasibility. For this study, acceptability will be defined as participants attending at least 80% of sessions. Finally, to identify any specific challenges to scaling up the intervention in the future, an established project evaluation questionnaire developed by investigators at the University of Miami will be used to pinpoint any modifications needed for the design of a larger, confirmatory randomized control trial. The questionnaire is made up of a series of closed ended yes/no questions, rating scales, and open-ended questions allowing for more qualitative data on participants’ opinions of the specific components of the intervention to improve the overall feasibility of the study for future iterations of implementation.

### Study measures

Based on our exclusion criteria, participants will complete the Mini-Mental Status Exam (MMSE) [54] to recognize potential subjects with dementia or severe cognitive abnormalities. To establish baseline walking ability, eligible participants will be required to complete the 10-m walk test which has been shown to have not only excellent reliability when used in older adult populations, but also be comparable in validity with other longer measures [55]. Participants’ viral load will be ascertained to determine viral suppression which is defined as < 200 copies/mL [56]. Potential participants will self-confirm safe participation by not having an acute or severe medical illness.

### Cardiometabolic measurements

Just prior to baseline, blood draws will be completed at the university affiliated adult HIV outpatient clinic from which participant recruitment will take place. These blood draws will yield baseline data on participants’ levels of HDL-C, LDL-C, triglycerides, insulin, and HbA1c. Fasting blood samples will be drawn via venipuncture and stored at 4 °C until analysis could be completed. Homeostatic model assessment (HOMA) will be calculated, providing a measure of insulin resistance. All the samples will be evaluated by commercial laboratory services using commercially available enzyme-linked immunosorbent assays. Simultaneously, participant physical characteristics such as blood pressure and hip-to-waist circumference will be collected to complement the measures of glucose, insulin resistance, and blood lipid profile mentioned previously. All of these measurements of cardiometabolic risk will be collected immediately post-intervention, and 3 months post-intervention as well.

### Psychosocial functioning

Measures of psychosocial functioning will be collected at all three timepoints as well -baseline, post-intervention, and 3 months post-intervention. To measure depression symptom severity, participants will complete the Center for Epidemiologic Scale of Depression [57], 9-item Patient Health Questionnaire [58], the Perceived Stress Scale [59], and the 7-item Generalized Anxiety Disorder scale to ascertain anxiety symptom intensity [60]. Finally, participants’ perceived social support will be measured by the frequently used and validated Multidimensional Scale of Perceived Social Support [61].

### Additional measures

Additional measures will be administered to participants to gain more insight on factors of acculturation, physical activity, stigma, quality of life, and overall patient satisfaction. At baseline, participants will complete a demographics form, provide a list of current medications, and respond to the Bidimensional Acculturation Scale [62]. Additionally, at all three time points-baseline, post-intervention, and 3 months post-intervention—subjects will be asked to complete the Global Physical Activity Questionnaire [63], the 12-item Short Form health survey [64], and the HIV Stigma Scale [65] to measure their overall physical activity levels, general health-related quality of life, and HIV-related stigma participants face on a day-to-day basis. Finally, participants will complete a project evaluation questionnaire developed by the investigators to indicate individual satisfaction in the intervention.

### Sample size

The total sample size for this pilot study will be 18 older Latino men living with HIV. A sample size of 18 will allow researchers to conduct three separate intervention groups of 6 people each. Prior work conducted by the authors indicated that 6 participants per intervention group was the optimum size to generate social interaction between participants, guard against attrition, while still being manageable for the CHW [41]. Although small, this sample size is consistent with similar pilot studies focused on physical activity among older adults [43, 44] and will be large enough to establish feasibility of the intervention in a population of older Latino men living with HIV.

### Anticipated statistical analysis plan

**Fig. 2:**
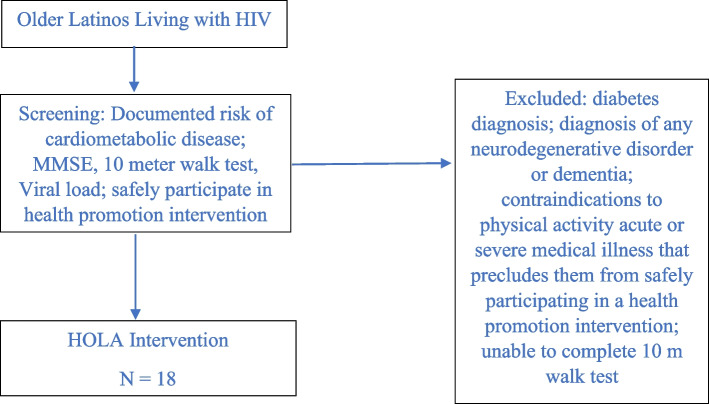
CONSORT diagram

In keeping with guidance from NIH funded biostatistical workgroups, this pilot study was not designed to be powered to test a hypothesis [42]. Therefore, since this study is designed to be a small pilot feasibility trial, best practices caution against using hypothesis testing in that they are likely to produce non-significant *p* values due to the study being underpowered. The CONSORT extension considers the use of descriptive statistics with 95% confidence intervals as more meaningful, an approach that parallels a similar structure employed in the first HOLA trial and comparable pilot trials [41, 53]. For variables continuous variables that are normally distributed, data will be analyzed and summarized using means and standard deviations. Alternatively, for continuous variables that are skewed, the median and range will be presented. Furthermore, data generated from categorical variables will be analyzed and summarized using counts and percentages (Fig. 2).

## Discussion

As the population of PLWH increasingly ages, high comorbidity between HIV and cardiometabolic diseases make older Latinos a high-risk population for whom innovative, scalable health promotion intervention could make a significant and lasting public health impact [19]. Despite the fact that older Latinos with HIV experience high levels of cardiometabolic disease due to issues associated with accelerated aging or potential adverse effects of ART medication, there is still a considerable dearth of relevant information on the effects of positive health promotion interventions in older Latinos living with HIV and comorbid cardiometabolic illness. Currently in preparation for a n adequately powered randomized control trial (e.g., securing grant funding), this study will generate evidence as to whether an already established health promotion intervention, HOLA, can be adapted for older Latino men with HIV to improve physical health, psychosocial functioning, and health-related quality of life in the midst of a rapid demographic transition. Authors are optimistic that this pilot project will yield significant results because this study builds upon prior work by authors delivering a health promotion intervention to prevent anxiety and depression in Latino older adults, a population living with high exposure to risk factors (comorbid physical and mental health conditions) and disparities in access to and engagement in mental health services [53].

The HOLA intervention is a multifaceted, innovative health promotion program that is uniquely tailored to meet the diverse needs and circumstances of older Latinos with HIV. Since many older adults who identify as racial/ethnic minorities hold stigmatizing views of mental health services [66, 67], HOLA builds on prior research and incorporates culturally relevant strategies to health promotion/behavior change as way of reducing mental and physical health risk factors among older Latinos with HIV [68]. Specifically, the use of a CHW to deliver a dual mental/physical health promotion intervention is an innovative approach to minimize disease burden in a population with high exposure to risk factors (in addition to HIV) and established disparities in both access to and engagement in beneficial mental and physical health services [69–71]. We believe that such an approach will appeal to older Latinos with HIV at risk for cardiometabolic disorders and psychological distress because of its non-stigmatizing presentation and the incorporation of cultural values/beliefs that promote the varying sociocultural influences contributing to the health of Latinos.

## Conclusions

High prevalence of HIV [2] combined with comorbid cardiometabolic diseases [7] makes older Latinos a high-risk population for whom scalable health promotion interventions could have great public health impact. Despite the high prevalence of cardiometabolic diseases in this population, there is a dearth of information on the effects of health promotion interventions on indices of cardiometabolic risk in midlife and older Latinos living with HIV. Thus, the study will provide valuable insight to evaluate the feasibility and acceptability of HOLA among HIV-infected Latinos aged 50 years and older and identify modifications needed in the design of a larger, ensuing hypothesis testing study. For example, there may be some challenges with attributing changes in cardiometabolic risk (blood pressure, fasting glucose, waist circumference) to the HOLA intervention using only three measurements in total over the intervention and post-intervention/maintenance phase. Some of these outcome measures may require more frequent measurement and others may change a very small amount over the short time period of the feasibility study. In addition, potential sustainability might be best measured by the drop-off after the last payment to the participants. With the evidence collected as part of the feasibility and acceptability trial, the authors plan to generate hypotheses concerning the intervention’s effects on cardiometabolic risk factors, psychosocial functioning, and health-related quality of life in a sample of midlife and older Latinos living with HIV.

## Data Availability

Not applicable.

## Abbreviations

HOLA: Happy Older Latinos are Active
HIV: Human immunodeficiency virus
CHW: Community health worker
USA: United States of America
ART: Antiretroviral therapy
MSM: Men who have sex with men
PWH: People with HIV
OAWH: Older adults with HIV
LSMM: Latino sexual minority men
SMM: Sexual minority men
MetS: Metabolic syndrome
NIH: National Institutes of Health
IRB: Internal Review Board
SLT: Social learning theory
BA: Behavioral activation
RA: Research assistant
MMSE: Mini-Mental Status Exam
mL: Milliliter
HDL-C: High-density lipoprotein cholesterol
LDL-C: Low-density lipoprotein cholesterol
HbA1c: Hemoglobin A1c
HOMA: Homeostatic Model Assessment
SPSS: Statistical Package for the Social Sciences
ANOVA: Analysis of variance

## Footnotes

1 The Hispanic Paradox is an epidemiological finding first described by Markides and Coreil (1986) to describe the puzzling fact that despite having lower average income and education, Latinos tend to have better health outcomes compared to their non-Latino White peers.

2 Task shifting involves the strategic redistribution of tasks among the healthcare team and personnel. Specific tasks are moved, shared or delegated, usually from highly trained health workers to those with shorter training or fewer qualifications, including lay people. This approach has been advocated as an important strategy to address healthcare resource shortages expand healthcare capacity.
